# Pathological Computed Tomography Features Associated With Adverse Outcomes After Mild Traumatic Brain Injury: A TRACK-TBI Study With External Validation in CENTER-TBI

**DOI:** 10.1001/jamaneurol.2021.2120

**Published:** 2021-07-19

**Authors:** Esther L. Yuh, Sonia Jain, Xiaoying Sun, Dana Pisică, Mark H. Harris, Sabrina R. Taylor, Amy J. Markowitz, Pratik Mukherjee, Jan Verheyden, Joseph T. Giacino, Harvey S. Levin, Michael McCrea, Murray B. Stein, Nancy R. Temkin, Ramon Diaz-Arrastia, Claudia S. Robertson, Hester F. Lingsma, David O. Okonkwo, Andrew I.R. Maas, Geoffrey T. Manley, and the TRACK-TBI Investigators for the CENTER-TBI Investigators, Opeolu Adeoye, Neeraj Badjatia, Kim Boase, Yelena Bodien, John D. Corrigan, Karen Crawford, Sureyya Dikmen, Ann-Christine Duhaime, Richard Ellenbogen, V. Ramana Feeser, Adam R. Ferguson, Brandon Foreman, Raquel Gardner, Etienne Gaudette, Luis Gonzalez, Shankar Gopinath, Rao Gullapalli, J. Claude Hemphill, Gillian Hotz, C. Dirk Keene, Joel Kramer, Natalie Kreitzer, Chris Lindsell, Joan Machamer, Christopher Madden, Alastair Martin, Thomas McAllister, Randall Merchant, Lindsay Nelson, Laura B. Ngwenya, Florence Noel, Amber Nolan, Eva Palacios, Daniel Perl, Miri Rabinowitz, Jonathan Rosand, Angelle Sander, Gabriella Satris, David Schnyer, Seth Seabury, Arthur Toga, Alex Valadka, Mary Vassar, Ross Zafonte

**Affiliations:** 1 Brain and Spinal Injury Center, San Francisco, California; 2 Department of Radiology and Biomedical Imaging, University of California, San Francisco, San Francisco; 3 Biostatistics Research Center, Herbert Wertheim School of Public Health and Human Longevity Science, University of California, San Diego, La Jolla; 4 Department of Neurosurgery, Erasmus Medical Center, Rotterdam, the Netherlands; 5 Department of Neurological Surgery, University of California, San Francisco, San Francisco; 6 Research and Development, Icometrix, Leuven, Belgium; 7 Department of Physical Medicine and Rehabilitation, Spaulding Rehabilitation Hospital, Charlestown, Massachusetts; 8 Department of Physical Medicine and Rehabilitation, Baylor College of Medicine, Houston, Texas; 9 Department of Neurosurgery, Medical College of Wisconsin, Milwaukee; 10 Department of Psychiatry, University of California San Diego, La Jolla; 11 Veterans Affairs San Diego Healthcare System, San Diego, California; 12 Department of Neurological Surgery, University of Washington, Seattle; 13 Department of Neurology, University of Pennsylvania, Philadelphia; 14 Department of Neurosurgery, Baylor College of Medicine, Houston, Texas; 15 Department of Public Health, Erasmus Medical Center, Rotterdam, the Netherlands; 16 Department of Neurological Surgery, University of Pittsburgh, Pittsburgh, Pennsylvania; 17 Department of Neurosurgery, Antwerp University Hospital and University of Antwerp, Edegem, Belgium; 18 Department of Physical Medicine and Rehabilitation, Harvard Medical School, Boston, Massachusetts; 19 University of Cincinnati, Cincinnati, Ohio; 20 University of Maryland, College Park; 21 University of Washington, Seattle; 22 Massachusetts General Hospital, Boston; 23 Ohio State University, Dublin; 24 University of Southern California, Los Angeles; 25 Massachusetts General Hospital for Children, Boston; 26 Virginia Commonwealth University, Richmond; 27 University of California, San Francisco, San Francisco; 28 TIRR Memorial Hermann, Houston, Texas; 29 Baylor College of Medicine, Houston, Texas; 30 University of Miami, Miami, Florida; 31 Vanderbilt University, Nashville, Tennessee; 32 UT Southwestern Medical Center, Dallas, Texas; 33 Indiana University, Bloomington; 34 Medical College of Wisconsin, Milwaukee; 35 Uniformed Services University, Bethesda, Maryland; 36 University of Pittsburgh, Pittsburgh, Pennsylvania; 37 Massachusetts General Hospital, Boston; 38 University of Texas at Austin, Austin; 39 Harvard Medical School, Boston, Massachusetts

## Abstract

**Importance:**

A head computed tomography (CT) with positive results for acute intracranial hemorrhage is the gold-standard diagnostic biomarker for acute traumatic brain injury (TBI). In moderate to severe TBI (Glasgow Coma Scale [GCS] scores 3-12), some CT features have been shown to be associated with outcomes. In mild TBI (mTBI; GCS scores 13-15), distribution and co-occurrence of pathological CT features and their prognostic importance are not well understood.

**Objective:**

To identify pathological CT features associated with adverse outcomes after mTBI.

**Design, Setting, and Participants:**

The longitudinal, observational Transforming Research and Clinical Knowledge in Traumatic Brain Injury (TRACK-TBI) study enrolled patients with TBI, including those 17 years and older with GCS scores of 13 to 15 who presented to emergency departments at 18 US level 1 trauma centers between February 26, 2014, and August 8, 2018, and underwent head CT imaging within 24 hours of TBI. Evaluations of CT imaging used TBI Common Data Elements. Glasgow Outcome Scale–Extended (GOSE) scores were assessed at 2 weeks and 3, 6, and 12 months postinjury. External validation of results was performed via the Collaborative European NeuroTrauma Effectiveness Research in Traumatic Brain Injury (CENTER-TBI) study. Data analyses were completed from February 2020 to February 2021.

**Exposures:**

Acute nonpenetrating head trauma.

**Main Outcomes and Measures:**

Frequency, co-occurrence, and clustering of CT features; incomplete recovery (GOSE scores <8 vs 8); and an unfavorable outcome (GOSE scores <5 vs ≥5) at 2 weeks and 3, 6, and 12 months.

**Results:**

In 1935 patients with mTBI (mean [SD] age, 41.5 [17.6] years; 1286 men [66.5%]) in the TRACK-TBI cohort and 2594 patients with mTBI (mean [SD] age, 51.8 [20.3] years; 1658 men [63.9%]) in an external validation cohort, hierarchical cluster analysis identified 3 major clusters of CT features: contusion, subarachnoid hemorrhage, and/or subdural hematoma; intraventricular and/or petechial hemorrhage; and epidural hematoma. Contusion, subarachnoid hemorrhage, and/or subdural hematoma features were associated with incomplete recovery (odds ratios [ORs] for GOSE scores <8 at 1 year: TRACK-TBI, 1.80 [95% CI, 1.39-2.33]; CENTER-TBI, 2.73 [95% CI, 2.18-3.41]) and greater degrees of unfavorable outcomes (ORs for GOSE scores <5 at 1 year: TRACK-TBI, 3.23 [95% CI, 1.59-6.58]; CENTER-TBI, 1.68 [95% CI, 1.13-2.49]) out to 12 months after injury, but epidural hematoma was not. Intraventricular and/or petechial hemorrhage was associated with greater degrees of unfavorable outcomes up to 12 months after injury (eg, OR for GOSE scores <5 at 1 year in TRACK-TBI: 3.47 [95% CI, 1.66-7.26]). Some CT features were more strongly associated with outcomes than previously validated variables (eg, ORs for GOSE scores <5 at 1 year in TRACK-TBI: neuropsychiatric history, 1.43 [95% CI .98-2.10] vs contusion, subarachnoid hemorrhage, and/or subdural hematoma, 3.23 [95% CI 1.59-6.58]). Findings were externally validated in 2594 patients with mTBI enrolled in the CENTER-TBI study.

**Conclusions and Relevance:**

In this study, pathological CT features carried different prognostic implications after mTBI to 1 year postinjury. Some patterns of injury were associated with worse outcomes than others. These results support that patients with mTBI and these CT features need TBI-specific education and systematic follow-up.

## Key Points

**Question:**

Are different patterns of intracranial injury on head computed tomography associated with prognosis after mild traumatic brain injury (mTBI)?

**Findings:**

In this cohort study, subarachnoid hemorrhage, subdural hematoma, and contusion often co-occurred and were associated with both incomplete recovery and more severe impairment out to 12 months after injury, while intraventricular and/or petechial hemorrhage co-occurred and were associated with more severe impairment up to 12 months after injury; epidural hematoma was associated with incomplete recovery at some points but not with more severe impairment. Some intracranial hemorrhage patterns were more strongly associated with outcomes than previously validated demographic and clinical variables.

**Meaning:**

In this study, different pathological features on head computed tomography carried different implications for mild traumatic brain injury prognosis to 1 year.

## Teaser

The longitudinal, observational study aims to identify pathological computed tomography features associated with adverse outcomes after mild traumatic brain injury.

## Introduction

A computed tomography (CT) with positive results for intracranial hemorrhage is the gold-standard diagnostic biomarker for acute TBI. Many (although not all) studies have shown that complicated mild TBI (mTBI), or mTBI with a positive head CT result, is associated with worse outcomes compared with uncomplicated mTBI. However, positive head CT results include a wide spectrum of intracranial lesions. A more precise understanding of the prognostic importance of CT abnormalities in mTBI, beyond the simple presence vs absence of abnormal findings on CT, is timely.

Associations between individual CT imaging features and outcomes have been demonstrated in moderate and severe TBI (Glasgow Coma Scale [GCS] scores 3-12).^1,2,3^ Similar efforts for mTBI have been stymied by subtler manifestations of impaired outcome, resulting in behavioral outcome measurements with less variability and a greater skew toward normal. Thus, a large study population is needed to accurately estimate the prognostic importance of individual CT features in patients with mTBI.

We used a large, longitudinal, observational cohort of patients with mTBI enrolled at US level 1 trauma centers for whom outcomes were measured at 2 weeks and 3, 6, and 12 months postinjury to determine the distribution and patterns of intracranial hemorrhage in mTBI and their implications for prognosis. We then externally validated these findings in a larger, independent, longitudinal observational cohort of patients with mTBI enrolled at European trauma centers.

## Methods

### Study Population

The Transforming Research and Clinical Knowledge in Traumatic Brain Injury (TRACK-TBI) study enrolled patients with TBI who presented to the emergency departments of 1 of 18 US level 1 trauma centers (eTable 1 in Supplement 1) and were treated along 1 of 3 care pathways (emergency department discharge, hospital admission without intensive care, or hospital admission with intensive care) (Table 1). The inclusion criterion for TRACK-TBI was presentation to a participating center within 24 hours of injury with a clinical indication for a head CT under American College of Emergency Medicine/US Centers for Disease Control and Prevention guidelines.^4^ Exclusion criteria included pregnancy, incarceration, nonsurvivable physical trauma, and preexisting medical or neuropsychiatric conditions that could interfere with outcome assessments. Institutional review boards of participating centers approved all study protocols. Patients or their legal representatives gave written informed consent. The Galveston Orientation and Amnesia Test was administered to determine ability to consent. For those without a passing score, a legally authorized representative gave initial consent and the competency screening was repeated at all follow-up visits. Race/ethnicity data (with options defined by the investigators) were collected to assess for racial/ethnic disparities in outcomes that have been reported in previous studies.^5,6,7^

**Table 1.** Demographic and Baseline Clinical Characteristics by Head Computed Tomography (CT) Status (n = 1935) in the Transforming Research and Clinical Knowledge in Traumatic Brain Injury Study

Characteristic	Total, No. (%)	Initial head CT with findings of acute intracranial abnormality, No. (%)		*P* value
		Negative	Positive	
Sex				
Male	1286 (66.5)	782 (60.8)	504 (39.2)	.004
Female	649 (33.5)	438 (67.5)	211 (32.5)	
Total	1935 (100.0)	1220 (63.0)	715 (37.0)	
Race				
White	1481 (77.5)	893 (60.3)	588 (39.7)	<.001
Black	318 (16.6)	244 (76.7)	74 (23.3)	
Other	113 (5.9)	71 (62.8)	42 (37.2)	
Total	1912 (100.0)	1208 (63.2)	704 (36.8)	
Hispanic ethnicity				
No	1526 (79.8)	969 (63.5)	557 (36.5)	.68
Yes	387 (20.2)	241 (62.3)	146 (37.7)	
Total	1913 (100.0)	1210 (63.3)	703 (36.7)	
Neuropsychiatric history				
No	1501 (77.7)	935 (62.3)	566 (37.7)	.24
Yes	432 (22.3)	283 (65.5)	149 (34.5)	
Total	1933 (100.0)	1218 (63.0)	715 (37.0)	
Prior traumatic brain injury				
Yes	586 (31.5)	409 (69.8)	177 (30.2)	<.001
No	1272 (68.5)	768 (60.4)	504 (39.6)	
Total	1858 (100.0)	1177 (63.3)	681 (36.7)	
Care pathway				
Emergency department discharge	503 (26.0)	453 (90.1)	50 (9.9)	<.001
Hospital admission without intensive care	833 (43.0)	584 (70.1)	249 (29.9)	
Hospital admission with intensive care	599 (31.0)	183 (30.6)	416 (69.4)	
Total	1935 (100.0)	1220 (63.0)	715 (37.0)	
Age, y				
Mean (SD)	41.5 (17.6)	37.7 (15.8)	47.8 (18.7)	<.001
Median (IQR) [range]	38 (26-55) [17-90]	34 (24-50) [17-88]	48 (31-64) [17-90]	
Education, y				
Mean (SD)	13.5 (2.9)	13.4 (2.7)	13.6 (3.2)	.046
Median (IQR) [range]	13 (12-16) [0-20]	12 (12-16) [1-20]	13 (12-16) [0-20]	

Abbreviation: IQR, interquartile range.

This article examines the subset of patients in the TRACK-TBI study who were 17 years or older at time of enrollment with GCS scores of 13 to 15 on emergency department arrival and an initial head CT available for review. eFigure 1 in Supplement 1 shows the recruitment and retention flowchart for the participants included in this analysis.

The Collaborative European NeuroTrauma Effectiveness Research in Traumatic Brain Injury (CENTER-TBI) study^8,9^ is a prospective, longitudinal, observational study of patients with TBI presenting to 1 of 55 trauma centers in Europe, with the same inclusion criteria and treatment along the same 3 care pathways as described for TRACK-TBI. The CENTER-TBI and TRACK-TBI studies are part of the International Initiative for TBI Research (https://intbir.incf.org/) and were codesigned for international collaboration.^10^

### CT Imaging and Evaluation of TBI Neuroimaging Common Data Elements

**Figure 1.**
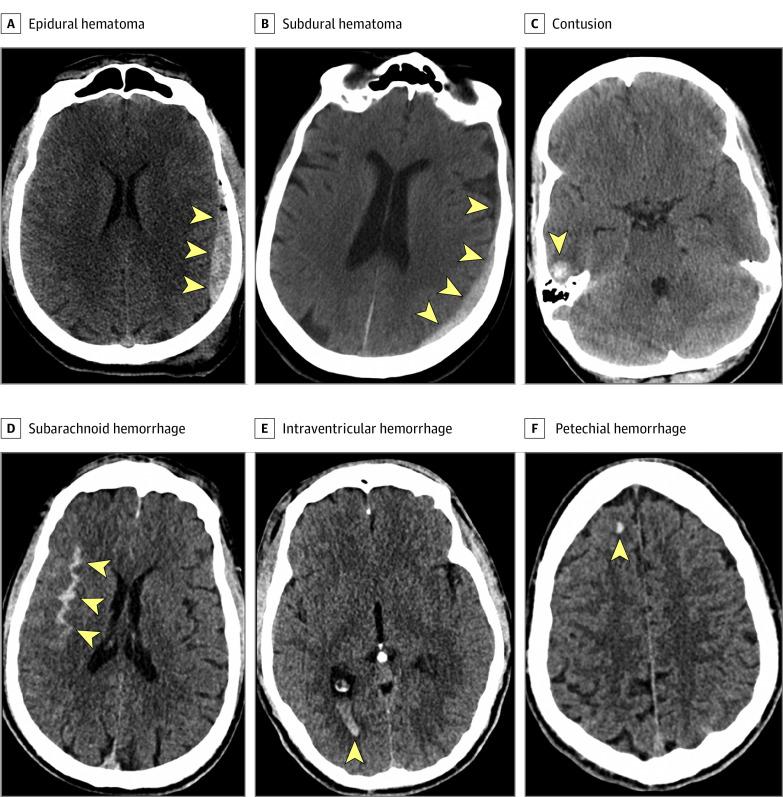
Examples of National Institute of Neurologic Disorders and Stroke Traumatic Brain Injury Neuroimaging Common Data Elements Corresponding to Different Subtypes of Acute Intracranial Hemorrhage Arrowheads indicate areas of intracranial hemorrhage.

In both TRACK-TBI and CENTER-TBI, the patients’ initial head CT images after injury were deidentified, uploaded to a central repository, and evaluated by a board-certified neuroradiologist (E.L.Y. and 1 nonauthor associated with the CENTER-TBI study) using National Institute of Neurological Disorders and Stroke (NINDS) TBI Neuroimaging Common Data Elements (CDEs).^11,12^ A positive CT result was defined as presence of any acute intracranial abnormality on the first head CT after admission, consistent with the US Food and Drug Administration definition.^13^ A positive CT result did not include an isolated skull fracture without an acute intracranial abnormality. The term *petechial hemorrhage* was used to describe small subcortical or deep hemorrhages that are the most common CT manifestation of the CDEs, traumatic axonal injury, and diffuse axonal injury. Readers (E.L.Y. and 1 nonauthor associated with the CENTER-TBI study) were blinded to clinical information except sex and age (and care path stratum, for CENTER-TBI). Figure 1 presents CDEs corresponding to different types of acute traumatic intracranial hemorrhage.

### Outcome Measure

The Glasgow Outcome Scale–Extended (GOSE) score is the most widely used measure of global functional outcome after TBI.^14,15,16^ In CENTER-TBI, the primary outcome measure was the GOSE score. In TRACK-TBI, the primary outcome measure was the GOSE-TBI score, which consists of the GOSE administered with the intent of specifically capturing disability associated with the TBI (ie, excluding any disability attributable to co-occurring traumas, such as orthopedic injuries).

### Statistical Analysis

Demographic, clinical, and CT characteristics were summarized descriptively. Between-group comparisons used Wilcoxon rank sum tests for continuous variables and Fisher exact tests for categorical variables were used. We used hierarchical cluster analysis (HCA) and multiple correspondence analysis (MCA) to derive CT phenotypes, or clusters of subtypes of intracranial hemorrhage, to mitigate potential multicollinearity issues.

Generalized estimating equation (GEE) models, a semiparametric approach to longitudinal analysis of correlated data, were used to study the association of demographics, clinical features, and CT features with incomplete recovery (GOSE scores <8 vs 8) and greater degrees of unfavorable outcome (GOSE scores <5 vs ≥5) at 2 weeks and 3, 6, and 12 months postinjury. The model included GOSE scores at each follow-up as the outcome; independent variables included demographics (age, sex, race/ethnicity, years of education), baseline clinical characteristics (prior TBI, neuropsychiatric history), CT clusters, data collection points (eg, 2 weeks), and interaction between CT clusters and data collection points. An unstructured working correlation matrix was used. We compared the marginal pseudo-*R*^2^ statistic for models to assess the contribution of CT variables.

We also performed GEE analysis to assess the association of the single most common CT pattern of intracranial injury, isolated subarachnoid hemorrhage (SAH), with incomplete recovery (GOSE scores <8 vs 8) and greater degrees of unfavorable outcome (GOSE scores <5 vs ≥5) at 2 weeks and 3, 6, and 12 months postinjury. Following complete analysis of the TRACK-TBI mTBI cohort, the same analytical approach and code used for the TRACK-TBI mTBI cohort analyses were applied to the CENTER-TBI mTBI cohort for external validation. All analyses were performed using R version 3.6.1 (R Foundation for Statistical Computing), using a threshold for statistical significance of *P* < .05, 2-tailed. Analyses were performed from February 2020 to February 2021.

## Results

### Demographic and Baseline Clinical Characteristics, CT Features, and GOSE

A total of 1935 individuals were eligible for the TRACK-TBI study. Of these, outcome measures (GOSE scores 1-8) (eTable 2 in Supplement 1) were available for 1497 (77.4%) at 2 weeks, 1381 (71.4%) at 3 months, 1311 (67.8%) at 6 months, and 1243 (64.2%) at 12 months. Table 1 presents demographic and baseline clinical characteristics of the TRACK-TBI cohort. Most participants were men (1286 [66.5%]). The most common clinical care pathway was hospital admission without an intensive care unit stay (833 [43.0%]). A positive head CT result was more likely in men (504 of 1286 men [39.2%]; 211 of 649 women [32.5%]; *P* = .004), individuals with higher education levels (mean [SD]: with positive CT results, 13.6 [3.2] years; with negative CT results, 13.4 [2.7] years; *P* = .046), and participants without a history of prior TBI (504 of 1272 participants without prior TBI [26.0%]; 177 of 586 participants with prior TBI [34.8%]; *P* < .001). A positive head CT result was less likely in Black individuals (74 of 318 Black individuals [23.3%]; 588 of 1481 White individuals [39.7%]; 42 of 113 individuals of other races [37.2%]; P<.001). There was no significant association with Hispanic ethnicity or history of neuropsychiatric disease.

**Figure 2.**
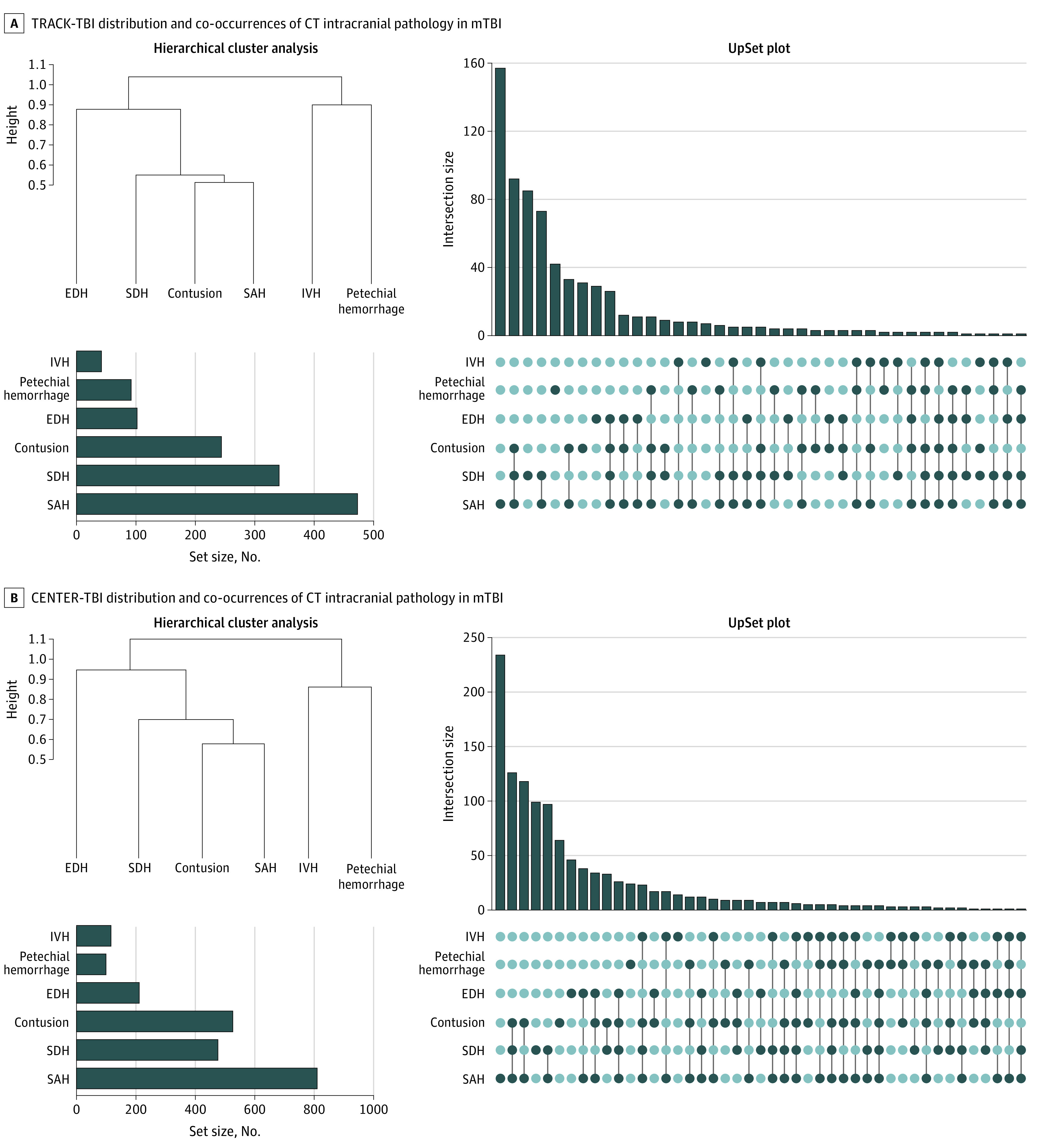
Distribution and Co-occurrences of Intracranial Pathology on Computed Tomography (CT) in Mild Traumatic Brain Injury (mTBI) by Cohort A, Distribution of National Institute of Neurological Disorders and Stroke TBI Neuroimaging Common Data Elements (CDEs) in participants 17 years and older with Glasgow Coma Scale scores of 13 to 15 (n = 1935) in the Transforming Research and Clinical Knowledge in TBI (TRACK-TBI) study. An UpSet plot shows that the most common pattern of acute intracranial hemorrhage is isolated subarachnoid hemorrhage (SAH), which constitutes 157 of 715 (22.0%) of all CT examinations showing intracranial hemorrhage. (Hierarchical cluster analysis demonstrates clusters of CT abnormalities. A dendrogram shows the distance at which the cluster was formed along the vertical axis, with 3 clusters: contusion, SAH, and/or subdural hematoma (SDH); intraventricular hemorrhage (IVH) and/or petechial hemorrhage; and epidural hemorrhage (EDH). The bar graph in the lower left corner shows that the most common acute intracranial abnormality was SAH (in 473 of 1935 patients [24.4%]), followed by SDH (341 [17.6%]), brain contusion (244 [12.6%]), EDH (102 [5.3%]), petechial hemorrhage (92 [4.8%]), and IVH (42 [2.2%]). B, Distribution of CDEs in participants 17 years and older with Glasgow Coma Scale scores of 13 to 15 (n = 2594) in the Collaborative European NeuroTrauma Effectiveness Research in Traumatic Brain Injury (CENTER-TBI) study. An UpSet plot shows that the most common pattern of acute intracranial hemorrhage is isolated SAH, which constitutes 234 of 1175 (19.9%) of all CT examinations positive for intracranial hemorrhage. Hierarchical cluster analysis shows clusters of CT abnormalities. A dendrogram shows the distance at which the cluster was formed along the vertical axis. The most common acute CT finding was SAH (810 of 2594 patients [31.2%]), followed by brain contusion (526 [20.3%]), SDH (476 [18.4%]), EDH (211 [8.1%], IVH (116 [4.5%]), and petechial hemorrhage (99 [3.8%]).

Figure 2A shows an UpSet plot of CT patterns of intracranial hemorrhage in descending order of frequency. Overall, 715 of 1935 individuals (37.0%) in this analytic cohort had a positive CT result for acute intracranial pathology. The most common pattern was isolated SAH (157 of 715 [22.0% of examinations with positive CT results]). Other common patterns were combined SAH, subdural hematoma (SDH), and contusion (92 examinations [12.9%]); isolated SDH (85 examinations [11.9%]); and combined SAH and SDH (73 examinations [10.2%]).

Figure 2A shows overall numbers of CT examinations with different acute intracranial hemorrhage subtypes. The most common was SAH (present in isolation or in combination with other findings on 473 CT examinations among all 1935 patients (24.4%), followed by SDH on 341 examinations (17.6%) and contusion on 244 examinations (12.6%). Less common were EDH on 102 examinations (5.3%), petechial hemorrhage on 92 examinations (4.8%), and intraventricular hemorrhage (IVH) on 42 examinations (2.2%).

The Rotterdam CT score^1^ (developed for moderate to severe TBI) demonstrated very minimal variability in this population of patients with mTBI. A total of 1873 of 1935 scores (96.8%) for the entire cohort were either 2 or 3.

### HCA and CT Phenotypes

Figure 2A shows results of HCA performed on CT intracranial hemorrhage subtypes in 1935 patients in the TRACK-TBI cohort. The dendrogram shows the existence of common clusters of CT abnormalities, or phenotypes. From the dendrogram and clinical experience, we define 3 clusters: (1) contusion, SAH, and/or SDH; (2) IVH and/or petechial hemorrhage; and (3) EDH. Multiple correspondence analysis recapitulated the HCA results, demonstrating identical groupings of CT findings (eFigure 2 in Supplement 1).

### Association of Demographics, Baseline Clinical Features, and CT Phenotypes With GOSE Scores Postinjury

We used GEE models to assess the association of demographics, baseline clinical features, and CT phenotypes with incomplete recovery (GOSE scores <8 vs 8; Table 2) and greater degrees of unfavorable outcomes (GOSE scores <5 vs ≥5; Table 3) at the 4 postinjury points. Regarding demographics and baseline clinical features, female sex (odds ratio [OR], 1.73 [95% CI, 1.43-2.08]; *P* < .001), neuropsychiatric history (OR, 1.61 [95% CI, 1.31-1.99]; *P* < .001), and TBI history (OR, 1.39 [95% CI, 1.16-1.67]; *P* < .001) were significantly associated with incomplete recovery (GOSE scores <8) but not greater degrees of unfavorable outcomes (GOSE scores <5). Age and fewer years of education were significantly associated with both incomplete recovery (GOSE scores <8; age [55 vs 26 years]: OR, 1.17 [95% CI, 1.00-1.37]; *P* = .04; education [16 vs 12 years]: OR, 0.64 [95% CI, 0.57-0.74]; *P* < .001) and greater degrees of unfavorable outcomes (GOSE scores <5; age [55 vs 26 years]: OR, 2.64 [95% CI, 2.02-3.46]; *P* < .001; education [16 vs 12 years]: OR, 0.60 [95% CI, 0.47-0.76]; *P* < .001).

**Table 2.** Associations of Demographic, Baseline Clinical, and Computed Tomography (CT) Phenotypes With Incomplete Recovery at 2 Weeks and 3, 6, and 12 Months Postinjury in the Transforming Research and Clinical Knowledge in Traumatic Brain Injury Study^a^

Variable	Odds ratio (95% CI)	*P* value
CT phenotypes		
Contusion, subarachnoid hemorrhage, and/or subdural hematoma		
2 wk	2.22 (1.61-3.06)	<.001
3 mo	1.87 (1.43-2.44)	<.001
6 mo	1.67 (1.28-2.17)	<.001
12 mo	1.80 (1.39-2.33)	<.001
Epidural hematoma		
2 wk	3.08 (1.27-7.49)	.01
3 mo	2.33 (1.28-4.24)	.006
6 mo	1.27 (0.74-2.17)	.39
12 mo	1.42 (0.85-2.37)	.18
Intraventricular and/or petechial hemorrhage		
2 wk	2.23 (1.10-4.51)	.03
3 mo	1.16 (0.69-1.93)	.58
6 mo	1.19 (0.74-1.92)	.46
12 mo	1.48 (0.92-2.38)	.10
Demographics		
Age (55 vs 26 y)^b^	1.17 (1.00-1.37)	.04
Years of education (16 vs 12 y)^b^	0.64 (0.57-0.74)	<.001
Sex (male vs female)	0.58 (0.48-0.70)	<.001
Race (White vs Black)	0.76 (0.59-0.98)	.09
Race (White vs other)^c^	1.22 (0.89-1.67)	.43
Ethnicity (Hispanic vs non-Hispanic)	1.11 (0.86-1.43)	.43
Baseline clinical characteristics		
Neuropsychiatric history (yes vs no)	1.61 (1.31-1.99)	<.001
Prior traumatic brain injury (yes vs no)	1.39 (1.16-1.67)	<.001

^a^ A generalized estimating equation model was used to study the association of demographic, clinical, and CT variables with incomplete recovery (Glasgow Outcome Scale–Extended [GOSE] scores <8 vs 8) at 2 weeks and 3, 6, and 12 months postinjury. The model included GOSE scores (<8 vs 8) at each follow-up as the outcome; independent variables included demographics, baseline clinical characteristics, CT phenotypes, data collection points (eg, 2 weeks), and interaction between CT phenotypes and data collection points. An unstructured working correlation matrix was used. The marginal *R*^2^ of the generalized estimating equation model was 9.1% without CT variables and 11.2% with CT variables.

^b^ For the continuous variables (age and years of education), we reported odds ratios comparing the third quartile vs the first quartile.

^c^ Other races includes Alaskan Native or Inuit, American Indian, Asian, Native Hawaiian or other Pacific Islander, and unknown categories.

**Table 3.** Associations of Demographic, Baseline Clinical, and Computed Tomography (CT) Features With Unfavorable Outcome at 2 Weeks and 3, 6, and 12 Months Postinjury in the Transforming Research and Clinical Knowledge in Traumatic Brain Injury Study^a^

Variable	Odds ratio (95% CI)	*P* value
CT phenotypes		
Contusion, subarachnoid hemorrhage, and/or subdural hematoma		
2 wk	2.14 (1.48-3.10)	<.001
3 mo	2.18 (1.23-3.89)	.008
6 mo	2.32 (1.23-4.38)	.01
12 mo	3.23 (1.59-6.58)	.001
Epidural hematoma		
2 wk	1.23 (0.58-2.64)	.59
3 mo	0.37 (0.08-1.64)	.19
6 mo	0.37 (0.08-1.62)	.19
12 mo	0.31 (0.06-1.70)	.18
Intraventricular and/or petechial hemorrhage		
2 wk	1.47 (0.82-2.62)	.19
3 mo	2.37 (1.14-4.92)	.02
6 mo	3.42 (1.62-7.22)	.001
12 mo	3.47 (1.66-7.26)	<.001
Demographics		
Age (55 vs 26 y)^b^	2.64 (2.02-3.46)	<.001
Years of education (16 vs 12 y)^b^	0.60 (0.47-0.76)	<.001
Sex (male vs female)	0.92 (0.64-1.31)	.63
Race (White vs Black)	0.90 (0.56-1.44)	.89
Race (White vs other)^c^	1.27 (0.60-2.69)	.81
Ethnicity (Hispanic vs non-Hispanic)	0.70 (0.39-1.28)	.25
Baseline clinical characteristics		
Neuropsychiatric history (yes vs no)	1.43 (0.98-2.10)	.07
Prior traumatic brain injury (yes vs no)	1.06 (0.73-1.53)	.78

^a^ A generalized estimating equation model was used to study the association of demographic, clinical, and CT variables with unfavorable outcome (Glasgow Outcome Scale–Extended [GOSE] scores <5 vs ≥5) at 2 weeks and 3, 6, and 12 months postinjury. The model included GOSE scores at each follow-up as the outcome; independent variables included demographic, baseline clinical characteristics, CT phenotypes, data collection points (eg, 2 weeks), and interaction between CT phenotypes and data collection points. An unstructured working correlation matrix was used. The marginal *R*^2^ of the generalized estimating equation model was 7.8% without CT variables and 10.0% with CT variables.

^b^ For the continuous variables (age and years of education), we reported odds ratios comparing the third quartile vs the first quartile.

^c^ Other races includes Alaskan Native or Inuit, American Indian, Asian, Native Hawaiian or other Pacific Islander, and unknown categories.

Regarding CT phenotypes derived from HCA or MCA, 3 trends emerged. The contusion, SAH, and/or SDH cluster was significantly associated with both incomplete recovery (ORs from 1.67 [95% CI, 1.28-2.17] at 6 months to 2.22 [95% CI, 1.61-3.06] at 2 weeks) and greater degrees of unfavorable outcomes (ORs from 2.14 [95% CI, 1.48-3.10] at 2 weeks to 3.23 [95% CI, 1.59-6.58] at 12 months) at all points from 2 weeks to 1 year. Epidural hematoma was associated only with incomplete recovery at earlier points (2 weeks and 3 months; ORs, 3.08 [95% CI, 1.27-7.49]; *P* = .01 and 2.33 [95% CI, 1.28-4.24]; *P* = .006, respectively) but not 6 or 12 months. Intraventricular and/or petechial hemorrhage was significantly associated with greater degrees of unfavorable outcomes at 3, 6, and 12 months (ORs, 2.37 [95% CI, 1.14-4.92]; 3.42 [95% CI, 1.62-7.22]; and 3.47 [95% CI, 1.66-7.26], respectively).

The marginal *R*^2^ of GEE models^17^ for incomplete recovery was 9.1% without CT variables and 11.2% with CT variables. The marginal *R*^2^ of GEE models for unfavorable outcomes was 7.8% without CT variables and 10.0% with CT variables.

We also performed post hoc GEE analysis of the association of isolated SAH (157 of 715 positive CT examination results [22.0%] for intracranial injury) with outcomes (eTables 3 and 4 in Supplement 1) and found significant association with incomplete recovery up to 6 months after injury (ORs: 2 weeks, 2.01 [95% CI, 1.19-3.39]; 3 months, 1.53 [95% CI, 1.00-2.35]; 6 months, 1.57 [95% CI, 1.01-2.43]). There was a trend toward significant association of isolated SAH with incomplete recovery at 12 months (OR, 1.36 [95% CI, 0.90-2.03]; *P* = .14).

When all 41 participants who underwent decompressive hemicraniotomy (21 for epidural hematoma) were excluded from the analytic cohort, odds ratios changed minimally (eTable 5 in Supplement 1 and Table 2). Finally, because patients with GCS scores of 13 may have worse prognoses than those with GCS scores of 14 or 15, we verified that the CT phenotypes remained prognostic for outcome up to 1 year after injury, even after participants with GCS scores of 13 were removed from the GEE models.

### External Validation in CENTER-TBI

A validation analysis was conducted in the CENTER-TBI cohort (n = 2594). As with TRACK-TBI, most participants in CENTER-TBI were men (1658 [63.9%] in CENTER-TBI vs 1286 of 1935 [66.5%] in TRACK-TBI) and had similar care pathways (eTable 6 in Supplement 1). The CENTER-TBI cohort had a higher incidence of positive CT findings (1175 of 2594 [45.3%] in CENTER-TBI vs 715 of 1935 [37.0%] in TRACK-TBI)), were older (mean [SD] ages, 51.8 [20.3] years vs 41.5 [17.6] years), and had a lower incidence of prior TBI (282 of 2494 participants with available TBI history [11.3%] in CENTER-TBI vs 586 of 1858 [31.5%] in TRACK-TBI).

Isolated SAH was the most common pattern in CENTER-TBI, similar to TRACK-TBI (234 of 1175 CT examinations with positive findings [19.9%] in CENTER-TBI vs 157 of 1175 CT examinations with positive findings [22.0%] in CENTER-TBI), and combined SAH, SDH, and/or contusion the second most common (126 [10.7%] vs 92 [12.9%]). Isolated SDH was the third most common pattern in TRACK-TBI and fourth most common in CENTER-TBI. Overall, the top 4 common patterns in TRACK-TBI were within the top 5 common patterns in CENTER-TBI (Figure 2B). Hierarchical cluster analysis and MCA in CENTER-TBI reproduced nearly identical CT imaging phenotypes found in TRACK-TBI (Figure 2; eFigures 2 and 3 in Supplement 1).

The GEE models also demonstrated consistent findings across TRACK-TBI (Tables 2 and 3) and CENTER-TBI (eTables 7 and 8 in Supplement 1). In both studies, the contusion, SAH, and/or SDH phenotype demonstrated significant associations with both incomplete recovery and greater degrees of unfavorable outcome at all points up to 1 year (eg, ORs for GOSE scores <8 at 1 year: TRACK-TBI, 1.80 [95% CI, 1.39-2.33]; CENTER-TBI, 2.73 [95% CI, 2.18-3.41]; ORs for GOSE scores <5 at 1 year: TRACK-TBI, 3.23 [95% CI, 1.59-6.58]; CENTER-TBI, 1.68 [95% CI, 1.13-2.49]). Intraventricular and/or petechial hemorrhage was associated with greater levels of unfavorable outcome in both studies up to 1 year (ORs for GOSE scores <5 at 1 year: TRACK-TBI, 3.47 [95% CI, 1.66-7.26]; CENTER-TBI, 1.82 [95% CI, 1.00-3.29]) and incomplete recovery in CENTER-TBI at 1 year (OR, 1.71 [95% CI, 1.11-2.62]). Epidural hematoma was associated with incomplete recovery at 1 year in CENTER-TBI (OR, 1.55 [95% CI, 1.02-2.36]) and at 2 weeks (OR, 3.08 [95% CI, 1.27-7.49]) and 3 months (OR, 2.33 [95% CI, 1.28-4.24]) in TRACK-TBI but was not associated with greater levels of unfavorable outcome at any point in either study. The *R*^2^ of GEE models^17^ for incomplete recovery and greater degrees of unfavorable outcomes in CENTER-TBI were similar to those in TRACK-TBI (10.2% vs 11.2% for GOSE scores <8, and 11.0% vs 10.0% for GOSE scores <5).

## Discussion

Fewer than half of all patients with mTBI evaluated at US level 1 trauma centers, and only 39% of patients with mTBI and positive head CT findings, receive follow-up care, including such simple interventions as provision of TBI educational materials at the time of discharge.^18^ In this study, we determined and then externally validated the distribution, patterns, and (importantly) clinical significance of intracranial CT findings in a large longitudinal observational cohort of 1935 patients with mTBI enrolled at 18 US level 1 trauma centers. The study population was enriched for so-called complicated mTBI: 37% of participants demonstrated intracranial hemorrhage on head CT, while the mean positive head CT rate in US emergency departments is approximately 9%.^19^ This enrichment provided sufficient power to determine the prognostic importance of CT abnormalities at a more granular level than simply positive vs negative categories. These more granular CT findings can immediately aid in the triage to TBI-specific education and systematic follow-up of the nearly 5 million patients with mTBI evaluated annually in US emergency departments.^19^ We also demonstrate, to our knowledge for the first time, the existence of common CT patterns or phenotypes of intracranial injury in mTBI and show that these different phenotypes have varying implications for outcomes up to 1 year postinjury.

The external validation of the findings in an independent prospective longitudinal observational cohort of 2594 patients with mTBI enrolled at 55 European trauma centers confirms the fidelity of our results. There was striking replication of results across TRACK-TBI and CENTER-TBI: contusion, SAH, and SDH often co-occur and were strongly associated with adverse outcomes over a broad range of GOSE scores up to 1 year postinjury in both studies. Intraventricular and/or petechial hemorrhage was associated with greater degrees of unfavorable outcome (GOSE scores <5) up to 1 year postinjury in both studies. Epidural hemorrhage was associated with incomplete recovery (GOSE scores <8 vs 8) at 3 months in TRACK-TBI and 1 year in CENTER-TBI but had no significant association with greater degrees of unfavorable outcome at any point in either study. Finally, some CT patterns of injury were even more strongly associated with outcomes than known demographic and clinical variables (older age, female sex, fewer years of education, and neuropsychiatric history),^20,21^ the second of which were reconfirmed across both studies to be variables significantly associated with adverse outcome.

We observed several minimal differences between TRACK-TBI and CENTER-TBI results. Intraventricular hemorrhage and/or petechial hemorrhage was significantly associated with incomplete recovery at 1 year in CENTER-TBI but not in TRACK-TBI. This may be because of higher statistical power in CENTER-TBI, based on both its larger sample size (n = 2594 vs n = 1935) and higher rate of positive CT findings compared with TRACK-TBI (45% vs 37%). In addition, a history of prior TBI had an apparent protective association against an unfavorable outcome in CENTER-TBI, while it was associated with incomplete recovery in TRACK-TBI. This may be because of differences in how prior TBI was assessed: CENTER-TBI used a short series of questions regarding medical history, and TRACK-TBI used a TBI-CDE standardized procedure for eliciting lifetime history of TBI via a structured interview,^22^ which may have captured more prior TBI events.

Most prior studies of mTBI outcome have treated head CT results as a binary variable (ie, any finding of an acute traumatic intracranial abnormality).^21,23,24,25,26,27,28,29,30,31,32,33,34,35,36^ Although many studies have reported an association of head CT positive for any acute traumatic intracranial finding with poorer outcome,^24,29,30,31,33,35^ others have shown no association,^25,26,32^ a weak association that does not endure in multivariable models that include demographic and other clinical factors,^21,23,28,34^ an association at 3 months but not at 6 months,^27^ or even an association with a better outcome.^36^ Recently, van der Naalt et al^21,37^ found that CT abnormalities were not associated with the 6-month outcome in either an emergency department model based on baseline factors nor an emergency department–plus model that included additional information (indicators of emotional distress and coping mechanisms) collected at a 2-week postinjury visit.

The few studies that have considered more granular CT pathology have found that most or all individual CT features are insignificant in multivariable models of outcome after mTBI.^38,39,40^ In some cases, this may have been in part because of a smaller study sample. However, even recent large studies have demonstrated negative results. Jacobs et al^38^ found that CT characteristics were not associated with significant improvement in an outcome prediction model based on clinical variables alone in a 1998-2006 series of 1999 consecutive patients with mTBI at a level 1 trauma center in the Netherlands. Specifically, the addition of head CT results to a prognostic model based on demographic and clinical characteristics resulted in a nonsignificant increase in the area under the curve from 0.69 to 0.70. Based on our results, we believe that reduced power because of a smaller sample size and/or lower positive CT rate, in addition to covariances (collinearity) among CT features, likely masked the significance of individual CT features in these previously reported multivariable models. In addition, cumulative advances in CT technology have resulted in continuous improvements in CT image quality over the past decade. Computed tomography scanners at US trauma centers now typically have 64 to 320 detector rows and 360° gantry rotation times less than 0.3 seconds,^41^ making thin sections, high-resolution multiplanar reconstructions, and whole-head acquisition in less than 1 second the new modern standard of care in CT imaging. These changes have likely significantly improved the diagnostic accuracy of CT imaging biomarkers over the past decade.

Finally, we surmise that the CT phenotypes we have described using data-driven analytics (HCA and MCA) provide a window into mechanisms of injury. Subarachnoid hemorrhage, contusion, and SDH often occur in the same patient. We speculate that these may occur primarily in injury mechanisms with linear acceleration or deceleration. The intraventricular and petechial hemorrhage category likely represents injuries including a significant component of rotational acceleration or deceleration,^42^ with IVH representing more severe rotational forces causing injury to deep structures. Superficial petechial hemorrhages in the subcortical white matter (eg, superior frontal gyrus) are more common and may represent milder cases of rotational acceleration or deceleration. Finally, the association of EDH with relatively good outcome has been demonstrated in studies of patients with moderate to severe TBI.^1^ We redemonstrate this in mTBI, showing that EDH is associated with early incomplete recovery but not with greater degrees of unfavorable outcome at any point. We also demonstrate that traumatic SAH, in isolation or combination with other features, is strongly associated with outcome in mTBI.

### Limitations

We recognize several limitations of this analysis. TRACK-TBI had follow-up rates of 77% at 2 weeks, 71% at 3 months, 68% at 6 months, and 64% at 12 months. The distribution of outcomes in participants lost to follow-up may have differed from those who attended 1 or more follow-up appointments. We note, however, that the 37% rate of positive CT findings in the entire TRACK-TBI cohort (n = 1935) was not significantly different from the 38% rate of positive CT findings in participants who attended at least 1 follow-up appointment (n = 1602). Also, both TRACK-TBI and CENTER-TBI were observational cohort studies designed to enroll participants in 3 care pathways (Table 1), resulting in 74% of the TRACK-TBI cohort and 70% of the CENTER-TBI cohort being admitted to the hospital or intensive care unit. Indeed, the incidence of CT abnormalities (37% in TRACK-TBI and 45% in CENTER-TBI) is higher than in some prior studies of mTBI.^38,43,44^ However, the CT phenotypes identified using HCA and MCA should depend only on the distribution of pathoanatomic results on head CT examinations positive for injury and should be unaffected by any number of additional head CT examinations with normal results in the cohort. This was confirmed by nearly identical results for both HCA and MCA in TRACK-TBI and CENTER-TBI.

## Conclusions

It is anecdotally taught that in moderate and severe TBI, outcome is determined by what “the injury brings to the patient”^39^^(p92)^ while in mTBI it is what “the patient brings to the injury.”^39^^(p92)^ In this study, while reconfirming the importance of patient baseline characteristics in mTBI outcome, we demonstrate for the first time (to our knowledge) that different pathological subtypes of intracranial hemorrhage are not equivalent in their implications for prognosis. This finding of varying odds ratios for different subtypes of intracranial hemorrhage, including high odds ratios for IVH and petechial hemorrhage as markers for rotational injury, appears to be a new observation in mTBI, and it invites further validation. By demonstrating variability in prognostic implications of different pathoanatomic lesion types, we show that in mTBI, as in moderate and severe TBI, some poor outcomes are attributable to what “the injury brings to the patient.”^39^^(p92)^

Based on 2 large observational studies conducted on different continents, contusion, SAH, SDH, IVH, and petechial hemorrhage are associated with adverse outcomes across a broad range of GOSE scores up to 1 year after mTBI, while EDH is not. These routinely obtained imaging findings can be used to identify patients at risk for unfavorable outcomes and improve clinical trial design. Patients with mTBI and these CT features should be considered for TBI-specific education and systematic follow-up.

## Notes

Supplement 1. eTable 1. TRACK-TBI and Center-TBI Study Sites eTable 2. TRACK-TBI distribution of GOSE scores at 2 weeks, and at 3, 6, and 12 months for all 1602 subjects who attended at least 1 follow-up appointment eTable 3. TRACK-TBI GEE model to assess association between isolated SAH and incomplete recovery (GOSE<8 vs. =8) at 2 weeks, 3, 6 and 12 months postinjury eTable 4. TRACK-TBI GEE model to assess association between isolated SAH and unfavorable outcome (GOSE<5 vs. ≥5) at 2 weeks, 3, 6 and 12 months postinjury eTable 5. TRACK-TBI association of demographic, baseline clinical, and CT phenotypes with incomplete recovery (GOSE<8 vs. =8) at 2 weeks, 3, 6 and 12 months postinjury after excluding all 41 patients who underwent surgical evacuation of intracranial hematoma. eTable 6. CENTER-TBI demographic and baseline clinical characteristics by head CT status (N=2594) eTable 7. CENTER-TBI association of demographic, baseline clinical, and CT phenotypes with incomplete recovery (GOSE<8 vs. =8) at 3, 6 and 12 months postinjury. eTable 8. CENTER-TBI association of demographic, baseline clinical, and CT features with unfavorable outcome (GOSE<5 vs. ≥5) at 3, 6 and 12 months postinjury.eFigure 1. Recruitment and retention flowchart eFigure 1. Recruitment and retention flowchart eFigure 2. TRACK-TBI - Multiple Correspondence Analysis (MCA) of CT predictors eFigure 3. CENTER-TBI Multiple Correspondence Analysis (MCA) of CT predictors

Supplement 2. Nonauthor Collaborators and CENTER-TBI Investigators
