# Supplementary material for: Pathological Computed Tomography Features Associated With Adverse Outcomes After Mild Traumatic Brain Injury: A TRACK-TBI Study With External Validation in CENTER-TBI
Source: JAMA Neurol. 2021 Jul 19;78(9):1–12. doi: 10.1001/jamaneurol.2021.2120 (PMC8290344; doi:10.1001/jamaneurol.2021.2120)
Supplement: Supplement 1. — eTable 1. TRACK-TBI and Center-TBI Study Sites eTable 2. TRACK-TBI distribution of GOSE scores at 2 weeks, and at 3, 6, and 12 months for all 1602 subjects who attended at least 1 follow-up appointment eTable 3. TRACK-TBI GEE model to assess association between isolated SAH and incomplete recovery (GOSE<8 vs. =8) at 2 weeks, 3, 6 and 12 months postinjury eTable 4. TRACK-TBI GEE model to assess association between isolated SAH and unfavorable outcome (GOSE<5 vs. ≥5) at 2 weeks, 3, 6 and 12 months postinjury eTable 5. TRACK-TBI association of demographic, baseline clinical, and CT phenotypes with incomplete recovery (GOSE<8 vs. =8) at 2 weeks, 3, 6 and 12 months postinjury after excluding all 41 patients who underwent surgical evacuation of intracranial hematoma. eTable 6. CENTER-TBI demographic and baseline clinical characteristics by head CT status (N=2594) eTable 7. CENTER-TBI association of demographic, baseline clinical, and CT phenotypes with incomplete recovery (GOSE<8 vs. =8) at 3, 6 and 12 months postinjury. eTable 8. CENTER-TBI association of demographic, baseline clinical, and CT features with unfavorable outcome (GOSE<5 vs. ≥5) at 3, 6 and 12 months postinjury.eFigure 1. Recruitment and retention flowchart eFigure 1. Recruitment and retention flowchart eFigure 2. TRACK-TBI - Multiple Correspondence Analysis (MCA) of CT predictors eFigure 3. CENTER-TBI Multiple Correspondence Analysis (MCA) of CT predictors [file jamaneurol-e212120-s001.pdf]

Supplemental Online Content

Yuh EL, Jain S, Sun X, et al; Pathological computed tomography features associated with adverse outcomes after mild traumatic brain injury: a TRACK-TBI study with external validation in CENTER-TBI. *JAMA Neurol*. Published online July 19, 2021. doi:10.1001/jamaneurol.2021.2120

eTable 1. TRACK-TBI and Center-TBI Study Sites
eTable 2. TRACK-TBI distribution of GOSE scores at 2 weeks, and at 3, 6, and 12 months for all 1602 subjects who attended at least 1 follow-up appointment
eTable 3. TRACK-TBI GEE model to assess association between isolated SAH and incomplete recovery (GOSE<8 vs. =8) at 2 weeks, 3, 6 and 12 months postinjury
eTable 4. TRACK-TBI GEE model to assess association between isolated SAH and unfavorable outcome (GOSE<5 vs. □5) at 2 weeks, 3, 6 and 12 months postinjury
eTable 5. TRACK-TBI association of demographic, baseline clinical, and CT phenotypes with incomplete recovery (GOSE<8 vs. =8) at 2 weeks, 3, 6 and 12 months postinjury after excluding all 41 patients who underwent surgical evacuation of intracranial hematoma.
eTable 6. CENTER-TBI demographic and baseline clinical characteristics by head CT status (N=2594)
eTable 7. CENTER-TBI association of demographic, baseline clinical, and CT phenotypes with incomplete recovery (GOSE<8 vs. =8) at 3, 6 and 12 months postinjury.
eTable 8. CENTER-TBI association of demographic, baseline clinical, and CT features with unfavorable outcome (GOSE<5 vs. ≥5) at 3, 6 and 12 months postinjury.
eFigure 1. Recruitment and retention flowchart
eFigure 2. TRACK-TBI - Multiple Correspondence Analysis (MCA) of CT predictors
eFigure 3. CENTER-TBI Multiple Correspondence Analysis (MCA) of CT predictors

This supplemental material has been provided by the authors to give readers additional information about their work.

© 2021 Yuh EL et al. *JAMA Neurology.*

eTable 1. TRACK-TBI Study Sites
**Transforming Clinical Knowledge in Traumatic Brain Injury (TRACK-TBI) sites**
Ben Taub General Hospital in Houston, TX
Massachusetts General Hospital in Boston, MA
Zuckerberg San Francisco General in San Francisco, CA
University of Cincinnati Medical Center in Cincinnati, OH
R. Adams Cowley Shock Trauma Center in Baltimore, MD
Ryder Trauma Center in Miami, FL
University of Pittsburgh Medical Center in Pittsburgh, PA
Seton Medical Center in Austin, TX
Parkland Memorial Hospital in Dallas, TX
Harborview Medical Center in Seattle, WA
Virginia Commonwealth University Medical Center in Richmond, VA
University of Pennsylvania Health System in Philadelphia, PA
Emory Healthcare in Atlanta, GA
Medical College of Wisconsin in Milwaukee, WI
University of Utah Health in Salt Lake City, UT
Indiana University Health in Indianapolis, IN
Hennepin County Medical Center in Minneapolis, MN
Denver Health in Denver, CO
**Collaborative European NeuroTrauma effectiveness research in traumatic brain injury (CENTER-TBI) sites:** *Please see Maas et al.*[9]

© 2021 Yuh EL et al. *JAMA Neurology.*

eTable 2. TRACK-TBI distribution of GOSE scores at 2 weeks, and at 3, 6, and 12 months for all 1602 subjects who attended at least 1 follow-up appointment

	W02	M03	M06	M12
1	14 (0.94%)	19 (1.38%)	24 (1.83%)	29 (2.33%)
2	5 (0.33%)	0 (0%)	0 (0%)	0 (0%)
3	85 (5.68%)	16 (1.16%)	11 (0.84%)	7 (0.56%)
4	76 (5.08%)	22 (1.59%)	12 (0.92%)	8 (0.64%)
5	378 (25.25%)	172 (12.45%)	97 (7.4%)	84 (6.76%)
6	312 (20.84%)	267 (19.33%)	244 (18.61%)	199 (16.01%)
7	311 (20.77%)	381 (27.59%)	399 (30.43%)	327 (26.31%)
8	316 (21.11%)	504 (36.5%)	524 (39.97%)	589 (47.39%)
Total	1497 (100%)	1381 (100%)	1311 (100%)	1243 (100%)

© 2021 Yuh EL et al. *JAMA Neurology.*

eTable 3. TRACK-TBI GEE model to assess association between isolated SAH and incomplete recovery (GOSE<8 vs. =8) at 2 weeks, 3, 6 and 12 months postinjury

		OR	95% CI	p-value
Isolated SAH	Week 2	2.009	(1.192, 3.387)	0.009
	Month 3	1.529	(0.997, 2.346)	0.052
	Month 6	1.568	(1.011, 2.432)	0.045
	Month 12	1.355	(0.904, 2.029)	0.141

eTable 4. TRACK-TBI GEE model to assess association between isolated SAH and unfavorable outcome (GOSE<5 vs. ≥5) at 2 weeks, 3, 6 and 12 months postinjury

		OR	95% CI	p-value
Isolated SAH	Week 2	1.305	(0.683, 2.492)	0.420
	Month 3	0.476	(0.106, 2.144)	0.334
	Month 6	1.122	(0.317, 3.976)	0.859
	Month 12	0.410	(0.042, 4.016)	0.444

Generalized estimating equation (GEE) models were used to study the association of isolated SAH with incomplete recovery (GOSE<8 vs. =8) and with unfavorable outcome (GOSE<5 vs. ≥5) at 2 weeks, 3, 6, and 12 months postinjury. The models were adjusted for demographic and baseline clinical characteristics (age, gender, race/ethnicity, years of education, prior TBI, and neuropsychiatric history).

© 2021 Yuh EL et al. *JAMA Neurology.*

eTable 5. TRACK-TBI association of demographic, baseline clinical, and CT phenotypes with incomplete recovery (GOSE<8 vs. = 8) at 2 weeks, 3, 6 and 12 months postinjury *after excluding all 41 patients who underwent surgical evacuation of intracranial hematoma.* (GEE model for GOSE<5 after all 41 cases of hemicraniotomy/hemicraniectomy could not be created, since there was only 1 patient with EDH who had GOSE<5 at any timepoint between 3 months and 1 year.)

Predictors		OR	95% CI	p-value
**CT Phenotypes**				
Contusion/SAH/SDH	Week 2	2.125	(1.539, 2.935)	<0.001
	Month 3	1.771	(1.353, 2.317)	<0.001
	Month 6	1.627	(1.245, 2.125)	<0.001
	Month 12	1.735	(1.335, 2.256)	<0.001
EDH	Week 2	2.484	(1.019, 6.059)	0.045
	Month 3	2.269	(1.160, 4.437)	0.017
	Month 6	1.118	(0.609, 2.053)	0.719
	Month 12	1.243	(0.702, 2.200)	0.455
IVH/Petechial Hemorrhage	Week 2	2.191	(1.084, 4.428)	0.029
	Month 3	1.119	(0.665, 1.885)	0.672
	Month 6	1.126	(0.691, 1.834)	0.633
	Month 12	1.406	(0.864, 2.289)	0.171
**Demographics**				
Age (55 vs 26)		1.163	(0.997, 1.356)	0.054
Years of Education (16 vs 12)		0.652	(0.572, 0.743)	<0.001
Sex (Male vs Female)		0.583	(0.484, 0.703)	<0.001
Race (White vs Black)		0.761	(0.592, 0.979)	0.084
Race (White vs Other)		1.239	(0.905, 1.696)	0.375
Ethnicity (Hispanic vs Non-Hispanic)		1.089	(0.842, 1.406)	0.517
**Baseline Clinical Characteristics**				
Neuropsychiatric history (Yes vs No)		1.599	(1.296, 1.973)	<0.001
Prior TBI (Yes vs No)		1.358	(1.132, 1.629)	0.001

© 2021 Yuh EL et al. *JAMA Neurology.*

Generalized estimating equation (GEE) model was used to study the association of demographics, clinical, and CT predictors with incomplete recovery (GOSE<8 vs. =8) at 2 weeks, 3, 6, and 12 months postinjury *after excluding all 41 patients who underwent surgical*

eTable 6. CENTER-TBI demographic and baseline clinical characteristics by head CT status (N=2594)			
	**Initial head CT with acute intracranial abnormality**		**p-value**
	**CT negative**	**CT positive (row %)**	
**Gender**			
Male N = 1658 (63.9%)	878 (53.0%)	780 (47.0%)	0.019*
Female N = 936 (36.1%)	541 (57.8%)	395 (42.2%)	
**Total N = 2594**	**1419 (54.7%)**	**1175 (45.3%)**	

evacuation of intracranial hematoma. The model included GOSE (<8 vs. =8) at each follow-up as the outcome; predictors included demographics, baseline clinical characteristics, CT phenotypes, timepoints, and interaction between CT phenotypes and timepoints. An unstructured working correlation matrix was used. Note for the continuous predictors (age and years of education), we reported OR comparing its third quartile vs. first quartile. The marginal R^2 of the GEE model was 8.9% without CT predictors and 10.7% with CT predictors.

© 2021 Yuh EL et al. *JAMA Neurology.*

**Race**			
White N = 2448 (97.0%)	1344 (54.9%)	1104 (45.1%)	0.041*
Black N = 37 (1.5%)	28 (75.7%)	9 (24.3%)	
Other N = 39 (1.5%)	22 (56.4%)	17 (43.6%)	
**Total N = 2524**	**1394 (55.2%)**	**1130 (44.8%)**	
**Hispanic**			
No N = 2479 (98.2%)	1374 (55.4%)	1105 (44.6%)	0.188
Yes N = 45 (1.8%)	20 (44.4%)	25 (55.6%)	
**Total N = 2524**	**1394 (55.2%)**	**1130 (44.8%)**	
**Neuropsychiatric history**			
No N = 2197 (85.5%)	1207 (54.9%)	990 (45.1%)	0.94
Yes N = 372 (14.5%)	203 (54.6%)	169 (45.4%)	
**Total N = 2569**	**1410 (54.9%)**	**1159 (45.1%)**	
**Prior TBI**			
Yes N = 282 (11.3%)	188 (66.7%)	94 (33.3%)	<0.001***
No N = 2212 (88.7%)	1182 (53.4%)	1030 (46.6%)	
**Total N = 2494**	**1370 (54.9%)**	**1124 (45.1%)**	
**Care pathway**			
ED Discharge N = 772 (29.8%)	686 (88.9%)	86 (11.1%)	<0.001***
Hospital admission, no ICU N = 1211 (46.7%)	620 (51.2%)	591 (48.8%)	
Hospital admission with ICU N = 611 (23.6%)	113 (18.5%)	498 (81.5%)	
**Total N = 2594**	**1419 (54.7%)**	**1175 (45.3%)**	
**Age**<br>Mean ± S.D. (Overall 51.8 ± 20.3 years)<br>[Min, Q1, Median, Q3, Max] (Overall [17,35,53,68,96]	49.0 ± 20.3 years<br>[17, 30, 49, 65, 94]	55.2 ± 19.7 years<br>[17, 40, 58, 71, 96]	<0.001****
**Education**<br>Mean ± S.D. (Overall 13.5 ± 4.1)<br>[Min, Q1, Median, Q3, Max] (Overall [1,11,13,16,30])	13.7 ± 4.1 years<br>[1,11,13,16,30]	13.3 ± 4.1 years<br>[3,11,13,16,30]	0.021*

© 2021 Yuh EL et al. *JAMA Neurology.*

eTable 7. CENTER-TBI association of demographic, baseline clinical, and CT phenotypes with incomplete recovery (GOSE<8 vs. = 8) at 3, 6 and 12 months postinjury.

Predictors		OR	95% CI	p-value
**CT Phenotypes**				
Contusion/SAH/SDH	Month 3	2.165	(1.737, 2.699)	<0.001***
	Month 6	2.404	(1.940, 2.979)	<0.001***
	Month 12	2.725	(2.181, 3.405)	<0.001***
EDH	Month 3	1.529	(0.994, 2.351)	0.053
	Month 6	1.491	(0.980 2.271)	0.062
	Month 12	1.549	(1.015, 2.364)	0.043*
IVH/Petechial Hemorrhage	Month 3	1.525	(0.963, 2.417)	0.072
	Month 6	1.545	(0.997, 2.394)	0.051
	Month 12	1.707	(1.114, 2.617)	0.014*
**Demographics**				
Age (55 vs 26)		1.226	(1.072, 1.401)	0.003**
Years of Education (16 vs 12)		1.009	(0.929, 1.096)	0.835
Sex (Male vs Female)		0.78	(0.653, 0.931)	0.006**
Race (White vs Black)		0.439	(0.234, 0.824)	0.028*
Race (White vs Other)		0.759	(0.379, 1.518)	0.715
Ethnicity (Hispanic vs Non-Hispanic)		1.82	(0.939, 3.528)	0.076
**Baseline Clinical Characteristics**				
Neuropsychiatric history (Yes vs No)		2.032	(1.589, 2.599)	<0.001***
Prior TBI (Yes vs No)		1.052	(0.812, 1.363)	0.701

Generalized estimating equation (GEE) model was used to study the association of demographics, clinical, and CT predictors with incomplete recovery (GOSE<8 vs. =8) at 3, 6, and 12 months postinjury. The model included GOSE (<8 vs. =8) at each follow-up as the outcome; predictors included demographics, baseline clinical characteristics, CT phenotypes, timepoints, and interaction between CT phenotypes and timepoints. An unstructured working correlation matrix was used. Note for the continuous predictors (age and years of education), we reported OR comparing the third quartile vs. first quartile values taken from the TRACK-TBI sample distribution. The marginal R^2 of the GEE model was 5.0% without CT predictors and 10.2% with CT predictors.

© 2021 Yuh EL et al. *JAMA Neurology.*

eTable 8. CENTER-TBI association of demographic, baseline clinical, and CT features with unfavorable outcome (GOSE<5 vs. ≥5) at 3, 6 and 12 months postinjury.

Predictors		OR	95% CI	p-value
**CT Phenotypes**				
Contusion/SAH/SDH	Month 3	1.995	(1.443, 2.758)	<0.001***
	Month 6	1.638	(1.131, 2.37)	0.009**
	Month 12	1.675	(1.129, 2.486)	0.01**
EDH	Month 3	1.487	(0.8, 2.765)	0.21
	Month 6	1.192	(0.556, 2.554)	0.652
	Month 12	1.091	(0.461, 2.583)	0.844
IVH/Petechial Hemorrhage	Month 3	1.945	(1.165, 3.246)	0.011*
	Month 6	1.869	(1.054, 3.312)	0.032*
	Month 12	1.815	(1.001, 3.289)	0.049*
**Demographics**				
Age (55 vs 26)		3.193	(2.336, 4.364)	<0.001
Years of Education (16 vs 12)		0.797	(0.687, 0.925)	0.003
Sex (Male vs Female)		0.969	(0.71, 1.321)	0.841
Race (White vs Black)		0.423	(0.139, 1.284)	0.282
Race (White vs Other)		1.091	(0.161, 7.373)	0.996
Ethnicity (Hispanic vs Non-Hispanic)		1.021	(0.257, 4.064)	0.976
**Baseline Clinical Characteristics**				
Neuropsychiatric history (Yes vs No)		2.058	(1.41, 3.005)	<0.001***
Prior TBI (Yes vs No)		0.42	(0.229, 0.77)	0.005**

Generalized estimating equation (GEE) model was used to study the association of demographics, clinical, and CT predictors with unfavorable outcome (GOSE<5 vs. ≥5) at 3, 6, and 12 months postinjury. The model included GOSE (<5 vs. ≥5) at each follow-up as the outcome; predictors included demographics, baseline clinical characteristics, CT phenotypes, timepoints, and interaction between CT phenotypes and timepoints. An unstructured working correlation matrix was used. Note for the continuous predictors (age and years of education), we reported OR comparing the third quartile vs. first quartile values taken from the TRACK-TBI sample distribution. The marginal R^2 of the GEE model was 9.0% without CT predictors and 11.0% with CT predictors.

© 2021 Yuh EL et al. *JAMA Neurology.*

eFigure 1. Recruitment and retention flowchart

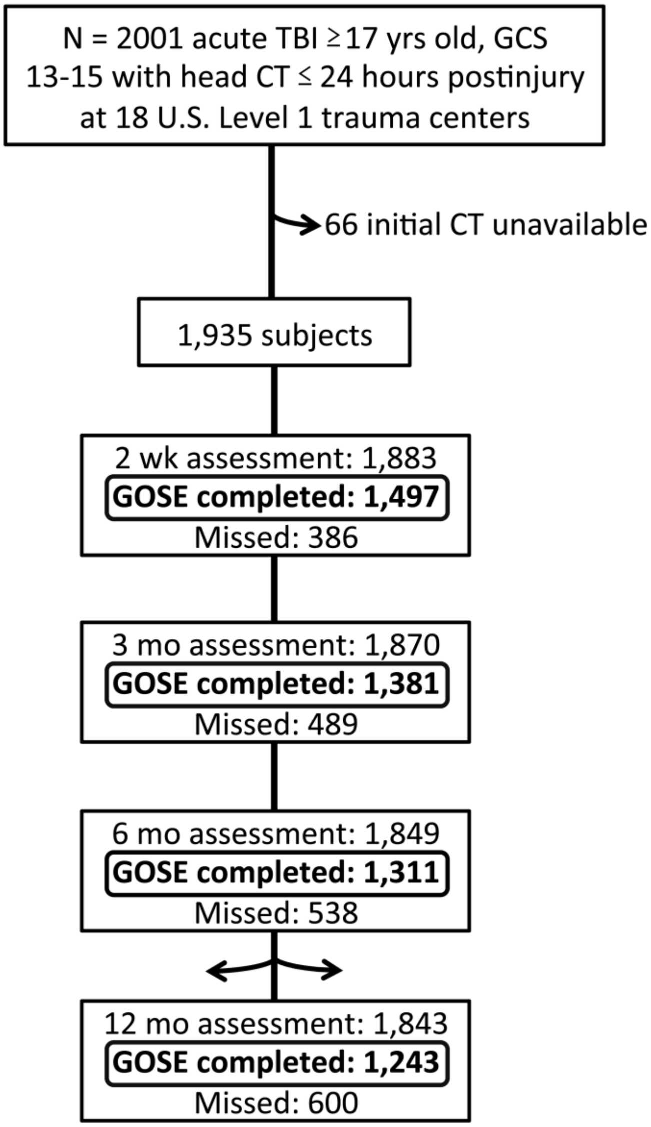

© 2021 Yuh EL et al. *JAMA Neurology.*

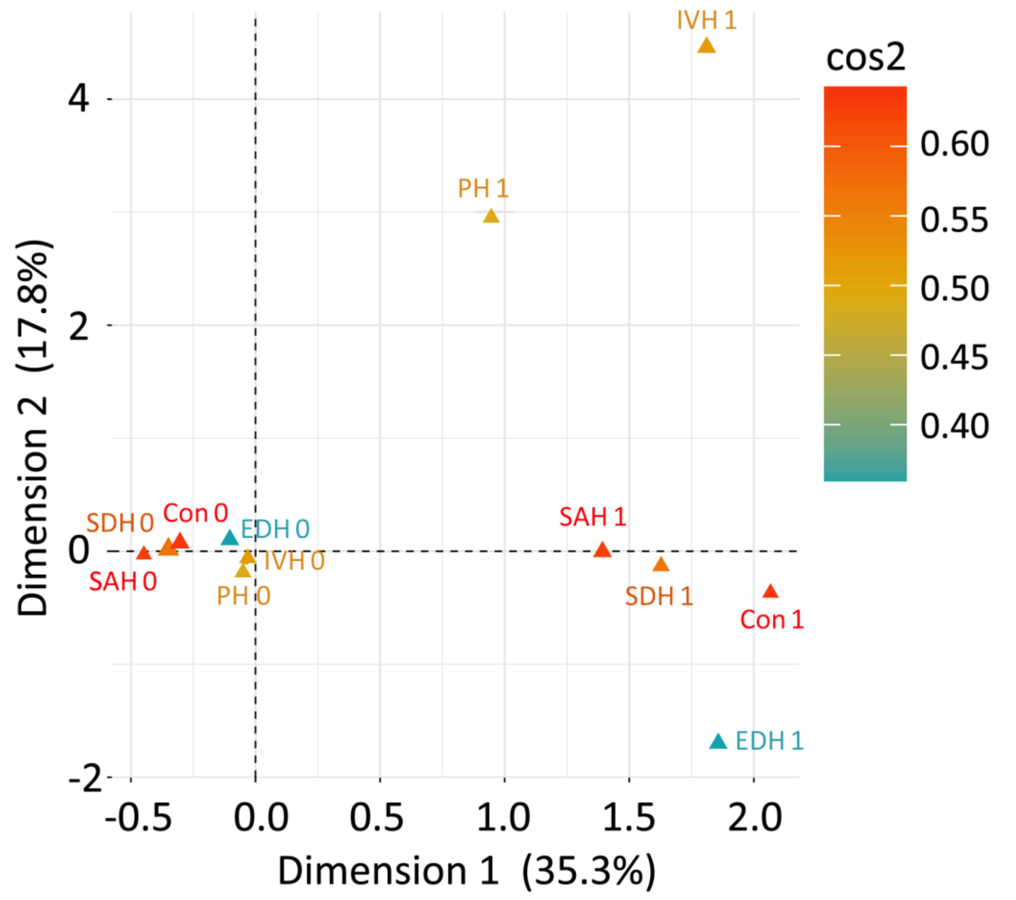

eFigure 2. TRACK-TBI - Multiple Correspondence Analysis (MCA) of CT predictors on all subjects (N=1935) recapitulates the hierarchical cluster analysis (HCA) results in the inset in the left upper corner of Figure 2. MCA shows the relationship between the CT categories. Categories with a similar profile are grouped together. The quality of representation (cos2) measures the degree of association between the CT categories and a particular axis. Con – contusion, EDH – epidural hematoma, IVH – intraventricular hemorrhage, PH – petechial hemorrhage, SAH – subarachnoid hemorrhage, SDH – subdural hematoma.

© 2021 Yuh EL et al. *JAMA Neurology.*

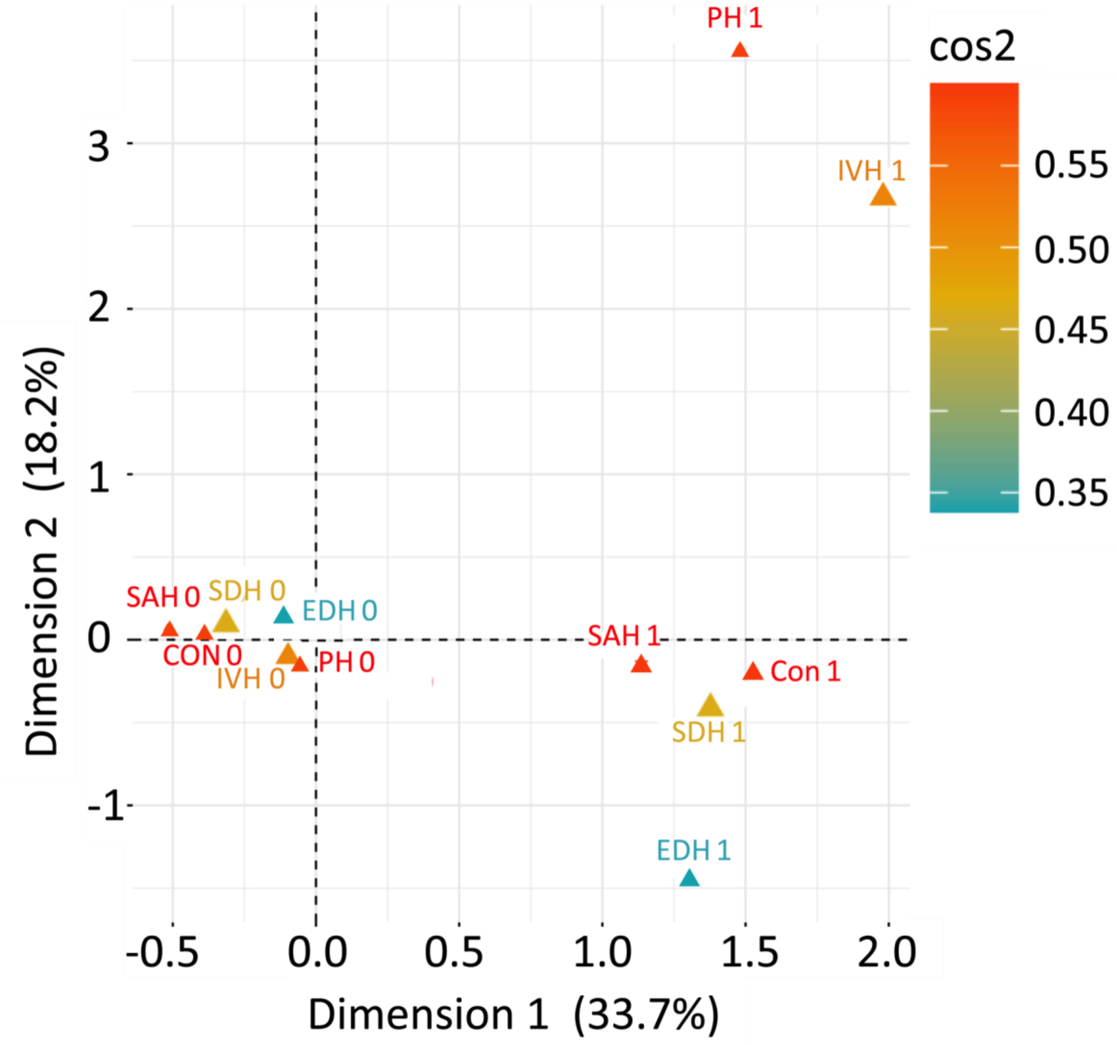

eFigure 3. CENTER-TBI Multiple Correspondence Analysis (MCA) of CT predictors on all subjects (N=2594) recapitulates the hierarchical cluster analysis (HCA) results in the inset in the left upper corner of eFigure 3. MCA shows the relationship between the CT categories. Categories with a similar profile are grouped together. The quality of representation (cos2) measures the degree of association between the CT categories and a particular axis. Con – contusion, EDH – epidural hematoma, IVH – intraventricular hemorrhage, PH – petechial hemorrhage, SAH – subarachnoid hemorrhage, SDH – subdural hematoma.

© 2021 Yuh EL et al. *JAMA Neurology.*
