# Supplementary material for: Pathological Computed Tomography Features Associated With Adverse Outcomes After Mild Traumatic Brain Injury: A TRACK-TBI Study With External Validation in CENTER-TBI
Source: JAMA Neurol. 2021 Jul 19;78(9):1–12. doi: 10.1001/jamaneurol.2021.2120 (PMC8290344; doi:10.1001/jamaneurol.2021.2120)
Supplement: Supplement 2. — Nonauthor Collaborators and CENTER-TBI Investigators [file jamaneurol-e212120-s002.pdf]

**Group Name(s): CENTER-TBI Investigators**							
**First Name and Middle Initial(s)**	**Last Name**	**Suffix (eg, Jr, III)**	Academic Degrees	Institution	Location (city, state/province, country)	Role or Contribution, eg, chair, principal investigator	Group (if more than 1 Group listed in the byline) and/or Subgroup (eg, Steering Committee)
Krisztina	Amrein						
Nada	Andelic						
Lasse	Andreassen						
Audny	Anke						
Anna	Antoni						
Gérard	Audibert						
Philippe	Azouvi						
Maria Luisa	Azzolini						
Ronald	Bartels						
Pál	Barzó						
Romuald	Beauvais						
Ronny	Beer						
Bo-Michael	Bellander						
Antonio	Belli						
Habib	Benal						
Maurizio	Berardino						
Luigi	Beretta						
Morten	Blaabjerg						
Peter	Bragge						
Alexandra	Brazinova						
Vibeke	Brinck						
Joanne	Brooker						
Camilla	Brorsson						
Andras	Buki						
Monika	Bullinger						
Manuel	Cabeleira						
Alessio	Caccioppola						
Emiliana	Calappi						

**First Name and Middle Initial(s)**	**Last Name**	**Suffix (eg, Jr, III)**	Academic Degrees	Institution	Location (city, state/province, country)	Role or Contribution, eg, chair, principal investigator	Group (if more than 1 Group listed in the byline) and/or Subgroup (eg, Steering Committee)
Maria Rosa	Calvi						
Peter	Cameron						
Guillermo	Carbayo Lozano						
Marco	Carbonara						
Ana M.	Castaño-León						
Simona	Cavallo						
Giorgio	Chevallard						
Arturo	Chieregato						
Giuseppe	Citerio						
Hans	Clusmann						
Mark	Coburn						
Jonathan	Coles						
Jamie D.	Cooper						
Marta	Correia						
Amra	Čović						
Nicola	Curry						
Endre	Czeiter						
Marek	Czosnyka						
Claire	Dahyot-Fizelier						
Paul	Dark						
Helen	Dawes						
Véronique	De Keyser						
Vincent	Degos						
Francesco	Della Corte						
Hugo	den Boogert						
Bart	Depreitere						
Đula	Đilvesi						
Abhishek	Dixit						

**First Name and Middle Initial(s)**	**Last Name**	**Suffix (eg, Jr, III)**	Academic Degrees	Institution	Location (city, state/province, country)	Role or Contribution, eg, chair, principal investigator	Group (if more than 1 Group listed in the byline) and/or Subgroup (eg, Steering Committee)
Emma	Donoghue						
Jens	Dreier						
Guy-Loup	Dulière						
Ari	Ercole						
Patrick	Esser						
Erzsébet	Ezer						
Martin	Fabricius						
Valery L.	Feigin						
Kelly	Foks						
Shirin	Frisvold						
Alex	Furmanov						
Pablo	Gagliardo						
Damien	Galanaud						
Dashiell	Gantner						
Guoyi	Gao						
Pradeep	George						
Alexandre	Ghuysen						
Lelde	Giga						
Ben	Glocker						
Jagoš	Golubovic						
Pedro A.	Gomez						
Johannes	Gratz						
Benjamin	Gravesteijn						
Francesca	Grossi						
Russell L.	Gruen						
Deepak	Gupta						
Juanita A.	Haagsma						
Iain	Haitsma						
Raimund	Helbok						

**First Name and Middle Initial(s)**	**Last Name**	**Suffix (eg, Jr, III)**	Academic Degrees	Institution	Location (city, state/province, country)	Role or Contribution, eg, chair, principal investigator	Group (if more than 1 Group listed in the byline) and/or Subgroup (eg, Steering Committee)
Eirik	Helseth						
Lindsay	Horton						
Jilske	Huijben						
Peter J.	Hutchinson						
Bram	Jacobs						
Stefan	Jankowski						
Mike	Jarrett						
Ji-yao	Jiang						
Faye	Johnson						
Kelly	Jones						
Mladen	Karan						
Cecilia	Kerlund						
Angelos G.	Kolias						
Erwin	Kompanje						
Daniel	Kondziella						
Evgenios	Kornaropoulos						
Lars-Owe	Koskinen						
Noémi	Kovács						
Ana	Kowark						
Alfonso	Lagares						
Linda	Lanyon						
Steven	Laureys						
Fiona	Lecky						
Didier	Ledoux						
Rolf	Lefering						
Valerie	Legrand						
Aurelie	Lejeune						
Leon	Levi						
Roger	Lightfoot						

**First Name and Middle Initial(s)**	**Last Name**	**Suffix (eg, Jr, III)**	Academic Degrees	Institution	Location (city, state/province, country)	Role or Contribution, eg, chair, principal investigator	Group (if more than 1 Group listed in the byline) and/or Subgroup (eg, Steering Committee)
Marc	Maegele						
Marek	Majdan						
Alex	Manara						
Costanza	Martino						
Hugues	Maréchal						
Julia	Mattern						
Catherine	McMahon						
Béla	Melegh						
David	Menon						
Tomas	Menovsky						
Ana	Mikolic						
Benoit	Misset						
Visakh	Muraleedharan						
Lynnette	Murray						
Ancuta	Negru						
David	Nelson						
Virginia	Newcombe						
Daan	Nieboer						
József	Nyirádi						
Otesile	Olubukola						
Matej	Oresic						
Fabrizio	Ortolano						
Aarno	Palotie						
Paul M.	Parizel						
Jean-François	Payen						
Natascha	Perera						
Vincent	Perlbarg						
Paolo	Persona						

**First Name and Middle Initial(s)**	**Last Name**	**Suffix (eg, Jr, III)**	Academic Degrees	Institution	Location (city, state/province, country)	Role or Contribution, eg, chair, principal investigator	Group (if more than 1 Group listed in the byline) and/or Subgroup (eg, Steering Committee)
Wilco	Peul						
Anna	Piippo-Karjalainen						
Matti	Pirinen						
Horia	Ples						
Suzanne	Polinder						
Inigo	Pomposo						
Jussi P.	Posti						
Louis	Puybasset						
Andreea	Radoi						
Arminas	Ragauskas						
Rahul	Raj						
Malinka	Rambadagalla						
Jonathan	Rhode						
Sylvia	Richardson						
Sophie	Richter						
Samuli	Ripatti						
Saulius	Rocka						
Cecilie	Roe						
Olav	Roise						
Jeffrey V.	Rosenfeld						
Christina	Rosenlund						
Guy	Rosenthal						
Rolf	Rossaint						
Sandra	Rossi						
Daniel	Rueckert						
Martin	Rusnák						
Juan	Sahuquillo						
Oliver	Sakowitz						

**First Name and Middle Initial(s)**	**Last Name**	**Suffix (eg, Jr, III)**	Academic Degrees	Institution	Location (city, state/province, country)	Role or Contribution, eg, chair, principal investigator	Group (if more than 1 Group listed in the byline) and/or Subgroup (eg, Steering Committee)
Renan	Sanchez-Porras						
Janos	Sandor						
Nadine	Schäfer						
Silke	Schmidt						
Herbert	Schoechl						
Guus	Schoonman						
Rico Frederik	Schou						
Elisabeth	Schwendenwein						
Charlie	Sewalt						
Toril	Skandsen						
Peter	Smielewski						
Abayomi	Sorinola						
Emmanuel	Stamatakis						
Simon	Stanworth						
Robert	Stevens						
William	Stewart						
Ewout W.	Steyerberg						
Nino	Stocchetti						
Nina	Sundström						
Riikka	Takala						
Viktória	Tamás						
Tomas	Tamosuitis						
Mark Steven	Taylor						
Braden	Te Ao						
Olli	Tenovuo						
Alice	Theadom						
Matt	Thomas						
Dick	Tibboel						

**First Name and Middle Initial(s)**	**Last Name**	**Suffix (eg, Jr, III)**	Academic Degrees	Institution	Location (city, state/province, country)	Role or Contribution, eg, chair, principal investigator	Group (if more than 1 Group listed in the byline) and/or Subgroup (eg, Steering Committee)
Marjolein	Timmers						
Christos	Tolias						
Tony	Trapani						
Cristina Maria	Tudora						
Andreas	Unterberg						
Peter	Vajkoczy						
Shirley	Vallance						
Egils	Valeinis						
Zoltán	Vámos						
Luc	van den Hauwe						
Mathieu	van der Jagt						
Joukje	van der Naalt						
Gregory	Van der Steen						
Jeroen T.J.M.	van Dijck						
Thomas A.	van Essen						
Wim	Van Hecke						
Caroline	van Heugten						
Dominique	Van Praag						
Thijs	Vande Vyvere						
Roel P. J.	van Wijk						
Alessia Vargiolu	van Wijk						
Emmanuel	Vega						
Kimberley	Velt						
Anne	Vik						
Rimantas	Vilcinis						
Victor	Volovici						
Nicole	von Steinbüchel						
Daphne	Voormolen						

**First Name and Middle Initial(s)**	**Last Name**	**Suffix (eg, Jr, III)**	Academic Degrees	Institution	Location (city, state/province, country)	Role or Contribution, eg, chair, principal investigator	Group (if more than 1 Group listed in the byline) and/or Subgroup (eg, Steering Committee)
Petar	Vulekovic						
Kevin K.W.	Wang						
Eveline	Wiegers						
Guy	Williams						
Lindsay	Wilson						
Stefan	Winzeck						
Stefan	Wolf						
Zhihui	Yang						
Peter	Ylén						
Alexander	Younsi						
Frederick A.	Zeiler						
Veronika	Zelinkova						
Agate	Ziverte						
Tommaso	Zoerle						
